# A case of right-sided Bochdalek hernia incidentally diagnosed in a gastric cancer patient

**DOI:** 10.1186/s12893-016-0145-2

**Published:** 2016-06-01

**Authors:** Satoru Kikuchi, Masahiko Nishizaki, Shinji Kuroda, Shunsuke Kagawa, Toshiyoshi Fujiwara

**Affiliations:** Department of Gastroenterological Surgery, Okayama University Graduate School of Medicine, Dentistry and Pharmaceutical Sciences, 2-5-1 Shikata-cho Kita-ku, Okayama, 700-8558 Japan

**Keywords:** Bochdalek hernia, Adult, Congenital diaphragmatic hernia, Right-sided, Laparoscopic surgery

## Abstract

**Background:**

Bochdalek hernia (BH) is generally congenital, presenting with respiratory distress. However, this pathology is rarely detected in adults. Some adult cases of BH present with symptoms attributed to the hernia, but incidental detection of BH is increasing among asymptomatic adults due to advances in imaging modalities. This report presents the management of incidental BH patients detected in the preoperative period of gastric cancer.

**Case presentation:**

An asymptomatic 76-year-old woman was diagnosed with advanced gastric cancer during follow-up after radiotherapy for uterine cervical cancer. Computed tomography (CT) was performed to exclude metastatic gastric cancer, incidentally detecting right-sided BH. We planned distal gastrectomy with lymph node dissection for gastric cancer and simultaneous repair of BH using a laparoscopic approach. We performed laparoscopic gastrectomy for gastric cancer and investigated the right-sided BH to assess whether repair during surgery was warranted. Herniation of the liver into the right hemithorax was observed, but was followed-up without surgical repair because the right hepatic lobe was adherent to the remnant right anterior hemidiaphragm and covered the huge defect in the right hemidiaphragm. No intra- or postoperative pneumothorax was observed during pneumoperitoneum.

**Conclusion:**

Regardless of symptoms, repair of adult BH is generally recommended to prevent visceral incarceration. However, BH in asymptomatic adults appears to be more common than previously reported in the literature. Surgeons need to consider the management of incidental BH encountered during thoracic or abdominal surgery.

## Background

Bochdalek hernia (BH) was first described in 1848 as a congenital hernia resulting from developmental failure of the posterolateral diaphragmatic formation to fuse properly [1]. Most BHs are diagnosed in the neonatal period with clinical symptoms caused by pulmonary insufficiency [2–4]. BH identified in adulthood is extremely rare and only around 100 cases of BH in adults have been reported. Moreover, only 35 cases of right-sided BH in adults have been reported [5, 6]. However, the prevalence of BH in adults has been estimated to range between 0.17 and 12.7 % [4, 7, 8]. Incidental identification of BH in asymptomatic adults is increasing due to advances in imaging modalities, and this pathology may be more common than previously reported. Adult BH patients are generally recommended to undergo surgical repair regardless of symptoms, to prevent the incarceration of viscera [9–11].

We report a case of gastric cancer with right-sided BH diagnosed incidentally on preoperative computed tomography (CT).

## Case presentation

**Fig. 1:**
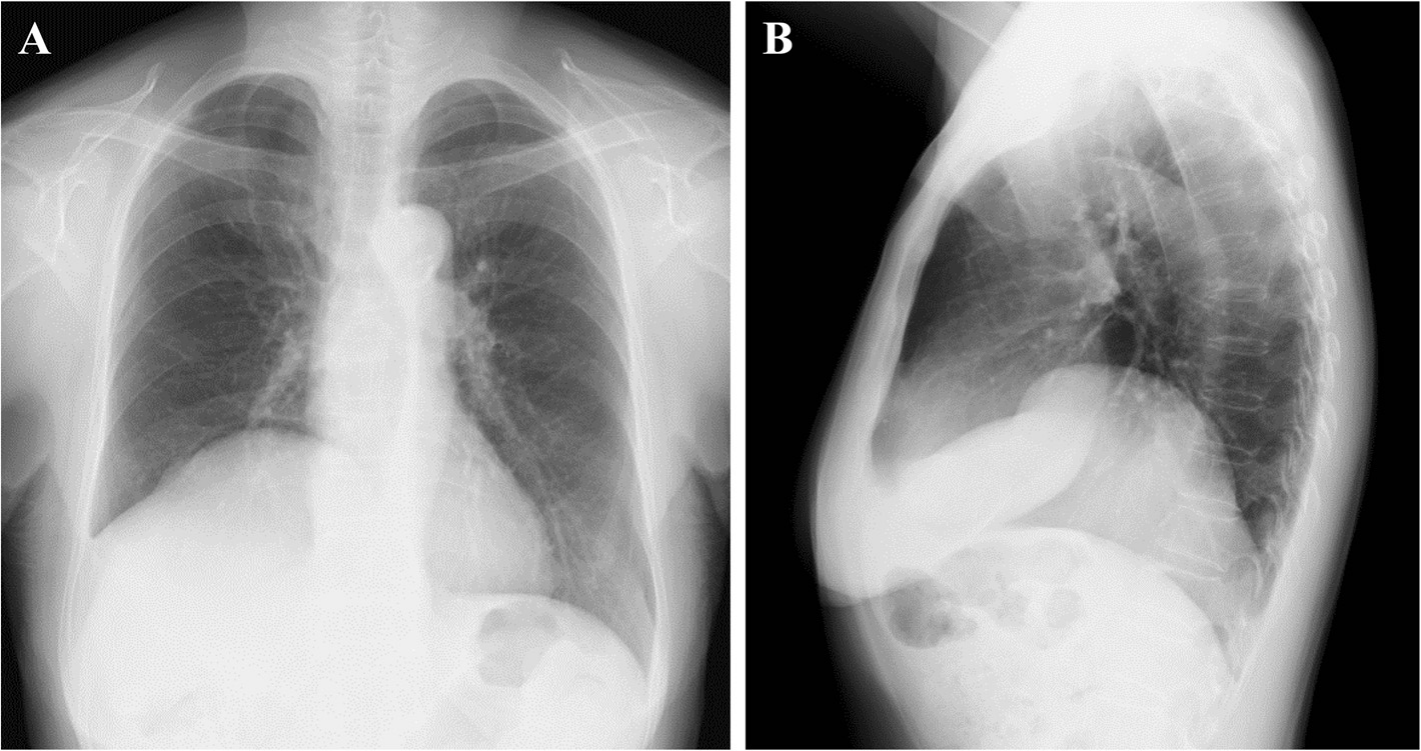
**a**, **b** Preoperative chest X-ray reveals an elevated right hemidiaphragm

**Fig. 2:**
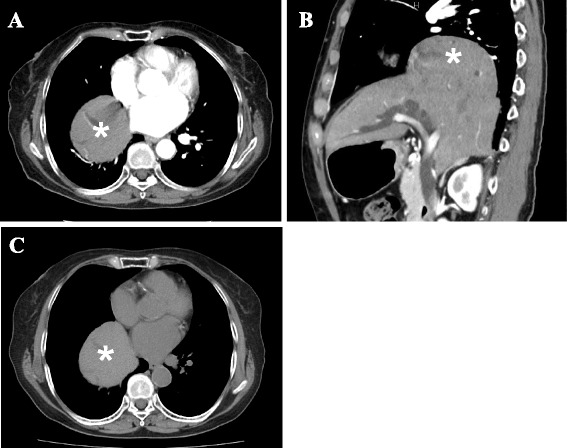
**a**, **b** Findings from preoperative thoracic and abdominal computed tomography (CT) for gastric cancer. Transverse CT shows the right hepatic lobe in the right inferior intrathoracic area (**a**: *asterisk*). On coronal section, the *asterisk* indicates the right hepatic lobe herniating into the right hemithorax via a diaphragmatic defect (**b**). **c** Thoracic and abdominal CT before radiotherapy for cervical cancer. The right hepatic lobe has herniated into the right hemithorax (*asterisk*), and appears no different in terms of the size of herniated liver compared to the preoperative CT for gastric cancer

**Fig. 3:**
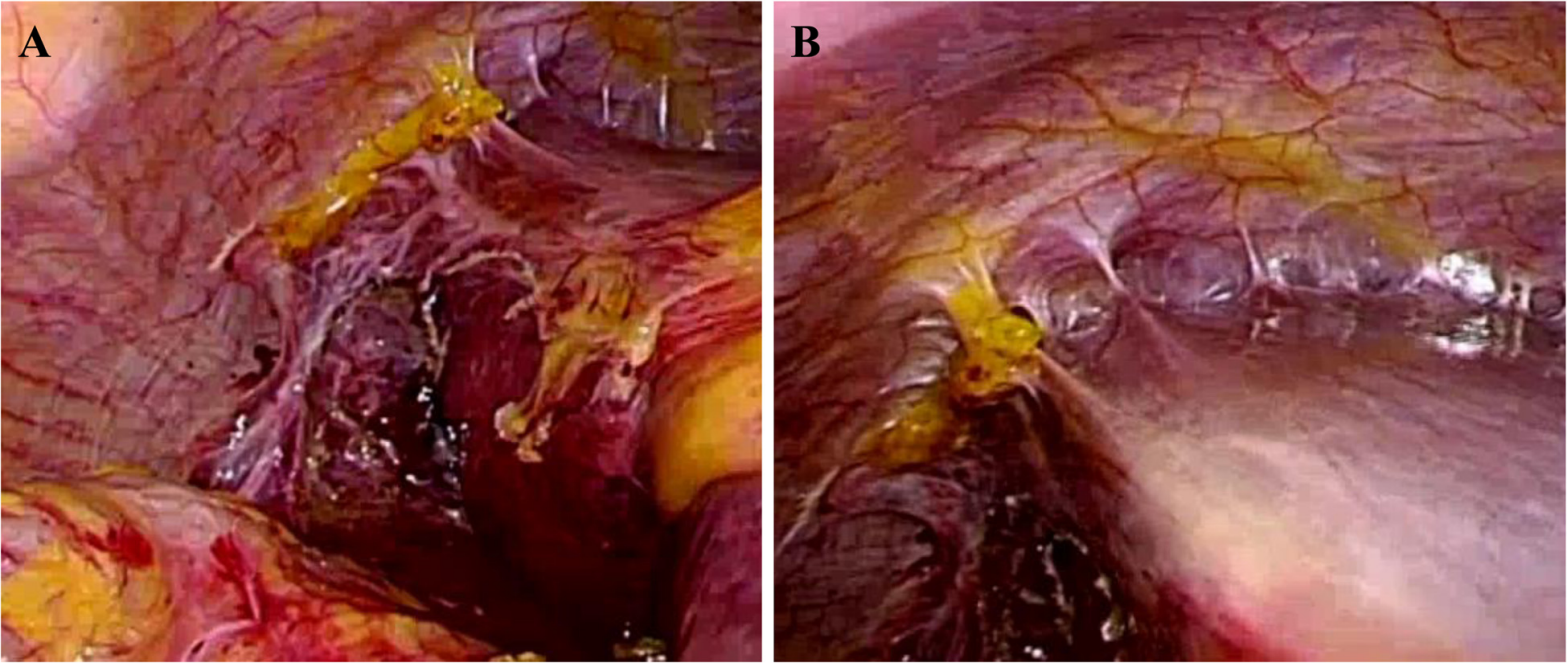
**a**, **b** Laparoscopic view of BH. The right hepatic lobe that had herniated into the thorax cavity is adherent to the remnant diaphragm and covers the diaphragm defect

An asymptomatic 76-year-old woman underwent follow-up fluorodeoxyglucose positron emission tomography (FDG-PET) after radiotherapy for uterine cervical cancer (T4N0M0 Stage IVA) and showed strong FDG accumulation in the stomach. She had no history of trauma. She had hypertension and cholangiectasis of unknown origin. Physical examination revealed nothing of note. Esophagogastroduodenoscopy (EGD) showed an elevated lesion, 30 mm in diameter, at the lesser curvature in the antrum of the stomach, and biopsy indicated well-differentiated adenocarcinoma. Chest X-ray revealed that the right hemidiaphragm was exceptionally high (Fig. 1a, b). Preoperative CT performed to rule out metastatic disease showed protrusion of the right hepatic lobe into the right hemithorax, and right-sided BH was therefore diagnosed incidentally (Fig. 2a, b). We re-evaluated images from CT performed 5 months earlier for the staging of the cervical cancer, to compare the condition of BH. No differences were apparent in terms of sizes of the defect or herniated liver between preoperative CT for gastric cancer and the CT for staging cervical cancer (Fig. 1a–c). The patient was diagnosed with advanced gastric cancer without lymph nodes or distant metastases (T2N0M0 Stage IB), accompanied by right-sided BH. We planned to perform distal gastrectomy with lymph nodes dissection for gastric cancer and simultaneous repair of the right-sided BH using the laparoscopic approach. Laparoscopic distal gastrectomy with lymph nodes dissection and cholecystectomy was performed under 10 cmH_2_O pressure of pneumoperitoneum. Intraoperatively, laparoscopic evaluation revealed herniation of the right hepatic lobe into the right hemithorax. However, we did not perform surgical repair of the BH because the right hepatic lobe that had herniated into the thoracic cavity was adherent to the remnant right anterior hemidiaphragm and covered the huge defect in the right hemidiaphragm (Fig. 3a, b). No intra- or postoperative pneumothorax was observed accompanying the pneumoperitoneum. The postoperative course was uneventful and the patient was discharged 14 days postoperatively. On follow-up at 3 months postoperatively, the patient was well and remained asymptomatic.

## Discussion

BH is a congenital diaphragmatic anomaly that occurs in one in 2000–12,500 live births, but is extremely rare in adults [10, 12]. This defect is caused by incomplete closure of the pleuroperitoneal folds around the 8th week of gestation. BH is more common on the left side (80–85 %), because complete closure occurs on the right side before the left side and the liver usually barricades against herniation on the right side. Most BHs are diagnosed in the neonatal period after the neonate presents with life-threating acute respiratory distress [11, 13]. In contrast to the acute presentation in neonatal BH, most adult BH patients present with more chronic symptoms, such as chest or abdominal pain, and 14 % of adult BHs are asymptomatic. Only 35 cases of right-sided BH in adults have been reported [5]. Although the true prevalence of adult BH remains unclear, Mullins reported that the incidence of adult BH was 0.17 %, with 68 % being right-sided and 77 % of patients being female, based on a review of 13,138 abdominal CT reports performed to rule out metastatic disease in patients with known malignant disease [7]. Those findings suggest that right-sided BH is more common than previously reported, and that female or right-sided BH may yield clinically silent disease. Although some cases of adult BH might be missed or unreported, incidental findings of adult BH seem likely to increase with the widespread use of advanced imaging modalities such as multi-detector row CT (MDCT) [14].

Chest X-rays may show abnormal contents above the diaphragm, and an air meniscus sign indicates the presence of BH [15]. However, BH may be difficult to appreciate on chest X-ray. In the current case, chest X-ray showed only elevation of the right hemidiaphragm (Fig. 1). CT and magnetic resonance imaging (MRI) are the most useful examinations for the diagnosis of BH. These modalities are suitable for detecting fat or soft tissue on the upper surface of the diaphragm and sagittal and coronal reformatted images can show the diaphragmatic defect and hernia contents. Another characteristic of BH is the posterolateral location. These findings were detected in the current case.

Most authors have suggested that BH in adults generally warrants surgical repair regardless of symptoms, given the risk of visceral incarceration [9, 10, 16], with the resulting defects repaired by primary repair or interposition of a mesh graft. Under emergency conditions, laparotomy has been considered the best approach for both left- and right-sided BH [6, 17, 18]. Under elective conditions, laparotomy or thoracotomy is performed for left-sided BH. Right-sided BH is generally dealt with using a thoracic or thoracoabdominal approach, because the right hepatic lobe obstructs an appropriate view to repair the defect [6]. Successful laparoscopic repair has recently been increasing for both left- and right-sided BH, even under emergency conditions [19–24]. In the current case, we detected an incidental right-sided BH before surgery for gastric cancer and planned to repair the right-sided BH concurrently with gastrectomy using a laparoscopic approach. The laparoscopic approach is minimally invasive and provides certain advantages over an open approach in terms of the surgical view to repair the defect, especially with the right-sided pathology, because the right hepatic lobe obstructs the surgical view and working space during diaphragmatic repair [20, 24]. We started laparoscopic gastrectomy under 10 cmH_2_O pressure of pneumoperitoneum. After creating pneumoperitoneum, no rise in airway pressure or decrease in pulse oximetry was seen, which indicated there was no tension pneumothorax in anesthesia. The surgery was subsequently continued using the same pneumoperitoneum pressure. During surgery, we observed the right-sided BH, and judged that repair was not needed because the right hepatic lobe that had herniated into the thorax cavity was adherent to the remnant right anterior hemidiaphragm and covered the huge defect in the right hemidiaphragm. Furthermore, the patient was asymptomatic and no progression of BH was seen between the preoperative CT for gastric cancer and CT before cervical cancer treatment. While adult BH is considered extremely rare and warrants repair regardless of symptoms, asymptomatic adult BH appears to be more common than previously reported. Surgeons should be aware of the presence of adult BH and consider whether repair is needed in the management of individual cases. A laparoscopic approach is useful to evaluate and repair BH, although surgeons have to be aware that intra- or postoperative pneumothorax can occur with pneumoperitoneum in this approach [25]. In the current case, no changes were observed on the monitor and vital signs remained stable intra- and postoperatively. No pneumothorax was observed on postoperative chest X-ray.

## Conclusion

In summary, BH in adults appears to be more common than previously thought, and incidental BHs are increasing due to advances in imaging modalities. Thoracic and abdominal surgeons should be aware of the potential presence of BH in adults, and consider the management of incidental BHs at the time of surgery in individual cases.

## Abbreviations

BH: Bochdalek hernia
CT: computed tomography
